# Phase Formation during the Synthesis of the MAB Phase from Mo-Al-B Mixtures in the Thermal Explosion Mode

**DOI:** 10.3390/ma17051025

**Published:** 2024-02-23

**Authors:** Artem Yu. Potanin, Evgeny A. Bashkirov, Dmitry Yu. Kovalev, Tatiana A. Sviridova, Evgeny A. Levashov

**Affiliations:** 1 National University of Science and Technology “MISIS”, Leninsky Prospect 4, bldg. 1, 119049 Moscow, Russia; a.potanin@inbox.ru (A.Y.P.); bashkirov.ea@misis.ru (E.A.B.); tim-17@yandex.ru (T.A.S.); 2 Merzhanov Institute of Structural Macrokinetics and Materials Science of the Russian Academy of Science, 142432 Chernogolovka, Russia; kovalev@ism.ac.ru

**Keywords:** self-propagating high-temperature synthesis (SHS), thermal explosion, MAB phase, MoAlB, time-resolved X-ray diffraction, mechanical activation

## Abstract

This work focused on the production of the MoAlB MAB phase through self-propagating, high-temperature synthesis in the thermal explosion mode. The influence of the method of a Mo-Al-B-powder reaction mixture preparation on the combustion temperature, mechanism, and stages of the MAB phase formation in the combustion process was investigated. The combustion temperatures of the mixtures obtained in the rotary ball mill and high-speed planetary ball mill were 1234 and 992 °C, respectively. The formation of intermediate compounds Mo_3_Al_8_ and α-MoB in the combustion front, along with MoAlB, was established using the time-resolved X-ray diffraction method. In the case of the mixture prepared in a ball mill, the primary interaction in the combustion front occurred through the Al melt, and in the case of using a planetary mill, solid-phase reactions played an important role. The mechanical activation of the mixture in a planetary mill also accelerated the processes of phase formation. The method of a reaction mixture preparation has virtually no effect on the MoAlB MAB phase content in combustion products (92–94%), but it does affect their structure. The synthesis products have a lamellar structure composed of MAB grains with a thickness of ~0.4 μm and a length of ~2–10 μm.

## 1. Introduction

Along with binary borides [1,2] (Mo_2_B, MoB, MoB_2_, Mo_2_B_5_, MoB_4_, and AlB_2_, AlB_12_) and intermetallics [3] (MoAl_12_, MoAl_5_, MoAl_4_, Mo_3_Al_8_, Mo_37_Al_63_, MoAl, and Mo_3_Al), the Mo-Al-B system also contains a single equiatomic ternary compound, MoAlB. The ternary phase diagram for the Mo-Al-B system is described in [4]. The MoAlB phase belongs to the family of MAB phases (where M is the early transition metal element, A is a group IIIA-VIA element, and B is boron) and is a class of layered (similar to the MAX phases [5]) ternary borides of transition metals [4]. This compound was first studied in 1942 by German scientists Halla and Thury and found to have the formula Mo_7_Al_6_B_7_ and to belong to the Pmmm space group [6]. In 1966, the structure of MoAlB was refined by Jeitschko [7]. MoAlB has an orthorhombic base-centered (oC) lattice (space group Cmcm) that consists of alternating MoB and Al layers [8]. The nanolaminated structure gives MoAlB some unique physical and mechanical properties, such as strength, crack resistance, and high electrical and thermal conductivities [8,9,10,11,12,13]. Furthermore, MoAlB ceramics exhibit fairly good oxidation resistance at temperatures above 1100 °C owing to the formation of a protective aluminum oxide layer on the surface [8,11,12,13,14].

MoAlB is the most studied material in the MAB phase family. Therefore, this composition has recently been drawing significant attention from researchers and has become one of the central objects of research and development in the field of ceramic materials. A quick and inexpensive procedure and a high content of the target phase are some of the requirements for MoAlB application. Over the past few years, researchers have been experimenting with various methods to fabricate this MAB phase, including reactive hot pressing [8], pressureless sintering [14], single-crystal growth from Al flux [15], salt melt synthesis [16], spark plasma sintering [17], and magnetron sputtering for thin film deposition [18]. Either (Mo, Al, B) elemental powders or a mixture of MoB boride and Al has been used as starting materials in experiments.

The self-propagating high-temperature synthesis (SHS) technique is a viable alternative for the production of the MAB phases. In this method, heat from the chemical reaction is consumed for heating the mixture and maintaining the combustion process, and unique structural states can be formed in the SHS wave [13,19,20]. The SHS process can be conducted in two main modes: layer-by-layer combustion and bulk combustion (thermal explosion) [19,20,21].

The thermal explosion mode frequently employed is Al-containing compounds [22]. The process involves heating the briquette to its autoignition point, at which the exothermic reaction proceeds simultaneously throughout the entire sample volume. The reaction between the elements often starts at a temperature close to the melting point of aluminum (T_Al_^melt^ = 660 °C). Both the MAX phases Ti_2_AlC [23], Ti_3_AlC_2_ [24], and Ti_2_AlN [25] and the MAB phases MoAlB [26], Fe_2_AlB_2_ [27], and Mn_2_AlB_2_ [28] have been synthesized under thermal explosion conditions. In refs. [26,29], mixtures of Mo/Al/B and Mo/Al/B_2_O_3_ powders were used as starting materials for the thermal explosion reaction to synthesize MoAlB.

In order to study the dynamics of real-time phase transitions during reactions, modern methods of crystal structure analysis using X-ray, neutron, and synchrotron radiations have been used [30,31,32,33,34,35,36]. These studies provide valuable insights into various materials’ phenomena. The stages of the MoAlB phase formation were studied for the layer-by-layer combustion and thermal explosion modes as reported in refs. [13,37]. Time-resolved X-ray diffraction data [13] indicate that the crystallization of the MoAlB phase is not accompanied by the formation of intermediate compounds. In the meantime, Merz et al. [37] discovered through neutron diffraction that in the case of rapid induction heating (~500 °C/min), the SHS process commences with the melting of aluminum. Subsequently, the MoAlB phase emerges following the transformation of β-MoB boride into α-MoB and the subsequent interaction with Al. Therefore, the MoAlB phase formation depends on the synthesis conditions and fabrication equipment, and the problem of studying the stages of the MoAlB phase formation still remains relevant.

A preliminary mechanical activation (MA) of the reaction mixture was performed to enhance the degree of conversion, the structural and phase homogeneity of combustion products, and, consequently, the content of various ternary compounds, including the MAX and MAB phases [38,39,40,41,42]. Due to the extensive plastic deformation processes, MA significantly reduces the heterogeneity of the reaction mixture, and energy is accumulated in structural defects in the reactants [43,44,45,46].

This study aimed to investigate the phase formation of synthesis products in the Mo-Al-B system during combustion synthesis in the thermal explosion mode, as well as to analyze the effect of the mechanical activation of the reaction mixture on the stages of chemical transformations.

## 2. Materials and Methods

Powders of molybdenum (PM-99.95 grade; size, 2–10 μm), aluminum (PA-4 grade; size, 20–200 μm), and black amorphous boron (B-99A grade; average particle size, 0.2 μm) were used as starting components. The reagents were mixed in an argon atmosphere using two types of mills in order to form MoAlB:

- A rotary ball mill (BM). Mixing was performed for 8 h at a jar rotational speed of 100 rpm;
- A high-speed planetary ball mill (PBM) “Activator-2SL” (Chemical Engineering Plant Ltd., Novosibirsk, Russia) [40,41]. Mixing was performed for 5 min at a jar rotational speed of 700 rpm.

An excess amount (4.5 wt.%) of aluminum powder was used to ensure that the elemental ratio in the mixture was stoichiometric. Briquettes sized 30 × 12.5 × 8 mm^3^ were compacted from the prepared mixtures to a relative density of 60% to conduct the experiments.

**Figure 1:**
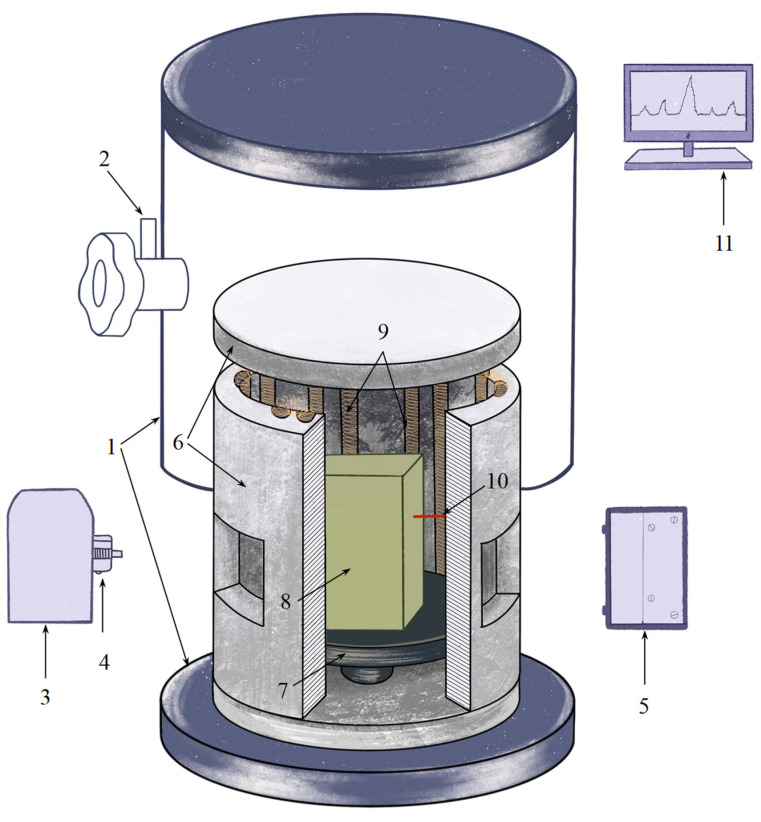
Diagram of the experimental TRXRD setup. 1—Reaction chamber; 2—Gas feed valve; 3—X-ray tube; 4—Monochromator; 5—Position-sensitive linear detector; 6—BN resistance furnace; 7—BN-Al_2_O_3_ platform; 8—Sample; 9—Heat coil; 10—Thermocouple; 11—Computer.

The stages of phase transformations in the combustion wave were studied using time-resolved X-ray diffraction (TRXRD) (Figure 1), which allows for the real-time detection of changes in the product composition [13,47]. This method consists of recording a series of X-ray diffraction patterns with minimal time exposure of the material during its heating. An in situ investigation of synthesis was conducted at all the stages, starting with the heating of the initial molded briquette, and ending with the cooling of the product. In this study, we used a setup based on the DRON diffractometer having a standard X-ray tube with a 2.5 kW power and a high-speed single-coordinate detector (exposure time of a single XRD pattern being ≥100 ms). The brilliance of the conventional X-ray tube was ~10^8^ photons/(s·mrad^2^·mm^2^·0.1%BW). The studies were conducted using monochromatic copper radiation (λ = 1.54178 Å); pyrolytic graphite was used as a monochromator. Horizontal reflection scanning in the Zeeman-Bolin geometry was employed.

The sample was placed in an airtight chamber mounted on a goniometer and equipped with a resistance furnace (Figure 1). It was heated using a built-in resistance furnace until autoignition occurred, and then the furnace was switched off. A collimated beam of radiation was directed at the central part of the sample surface at an angle of ~20° and illuminated an area of 2 × 10 mm^2^. The angular scan pitch was chosen to be 2θ = 30°–50° so that diffraction lines of the initial and resulting phases could be recorded. The exposure time of a single XRD pattern was 0.5 s; the number of XRD patterns in the series was 64. Up to three series of XRD patterns characterizing the phase formation process throughout the entire synthesis, until the products had been cooled down, were recorded.

A W–Re micro-thermocouple contacting the sample surface was used to measure the temperature. The signals from the thermocouple were recorded at a frequency of 250 Hz through an analog-to-digital converter and synchronized with the instant at which the XRD pattern of the process started being recorded. The studies were conducted in a helium atmosphere at an excessive pressure of 1.2 atm.

The structural studies were conducted using an S-3400N scanning electron microscope (SEM) (Hitachi, Tokyo, Japan) equipped with a NORAN X-ray system 7 energy-dispersive X-ray spectrometer (Thermo Scientific, Waltham, MA, USA) for the electron probe microanalysis of structural components. XRD phase analysis was carried out on a DRON-3 diffractometer (JSC Research Center “Burevestnik”, Saint Petersburg, Russia) using monochromatic Cu-Kα radiation. Stepwise scanning was conducted in the 2θ range = 10°–110° (scanning pitch, 0.05°; exposure time, 4 s per point). The recorded XRD patterns were analyzed using the software package [48], employing the simplified Rietveld refinement method (atomic coordinates were not varied) [49], which allowed us to determine volume fractions of the phases and their lattice parameters with an accuracy of 5–10% and 0.00010–0.00015 nm, respectively.

## 3. Results and Discussion

**Figure 2:**
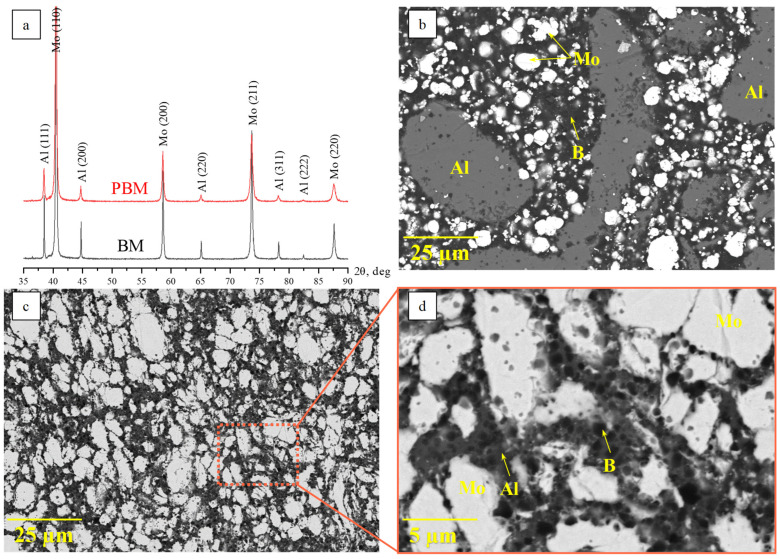
X-ray diffraction patterns (**a**) and the morphology of reaction mixtures after treatment in the BM (**b**) and PBM (**c**,**d**).

An analysis of the microstructures and XRD patterns of the Mo-Al-B reaction mixtures milled in the BM and PBM shows that a high-intensity mechanical activation significantly affected the material structure (Figure 2). In the mixture prepared in the BM, the shape and size of the initial reagents remained virtually unchanged during mixing (Figure 2b). The mixture consisted of coarse aluminum particles with an average size of 50–100 μm, fine rounded molybdenum particles sized up to 10 μm, and highly dispersed agglomerated boron. High-energy ball milling (HEBM) in the PBM alters the size and morphology of reagent particles (Figure 2c,d). Aluminum particles become significantly comminuted, while molybdenum particles become coarser. In these HEBM modes, boron particles are deagglomerated and distributed uniformly over the surface and volume of metal particles. XRD phase analysis demonstrated that the phase composition was not altered during the reaction mixture preparation, and that no products of mechanochemical synthesis were formed (Figure 2a).

**Figure 3:**
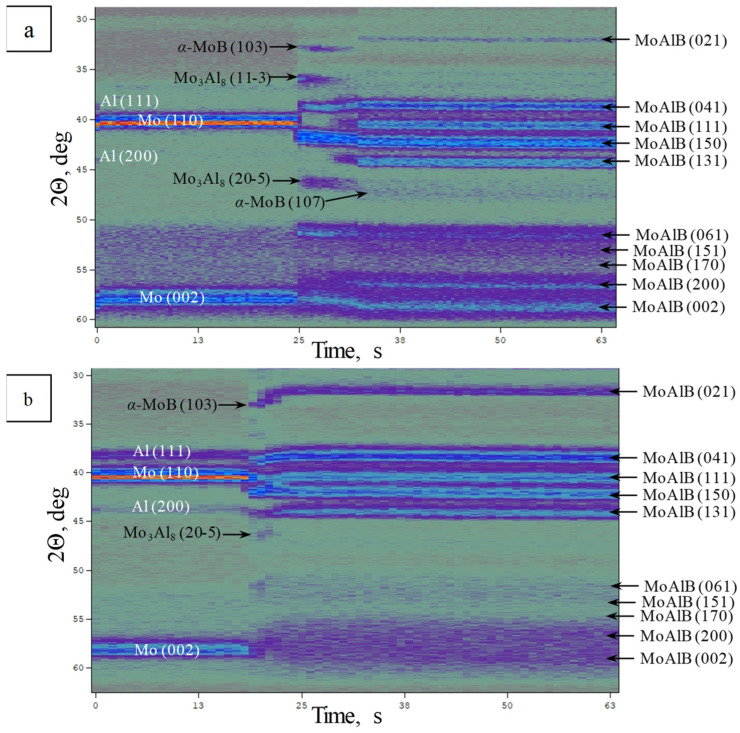
The TRXRD pattern of phase formation for MoAlB from a mixture prepared in an BM (**a**) and a PBM (**b**) in the mode of thermal explosion.

Figure 3 shows the diffraction pattern of the phase formation during the thermal explosion of Mo-Al-B mixtures. A series of XRD patterns is shown as a two-dimensional field in angle vs. time coordinates, and the diffraction line intensity is depicted by the color of the field. An analysis of the diffraction field demonstrated that the phase formation involved several stages in both cases. The intensities of the initial elements’ diffraction lines abruptly decreased during synthesis, and their positions were shifted to smaller angles due to the thermal expansion of the crystal lattice. Reflections corresponding to the MoAlB’s main phase, as well as the intermediate α-MoB and Mo_3_Al_8_ phases, emerged after the diffraction lines belonging to the initial components had disappeared.

**Figure 4:**
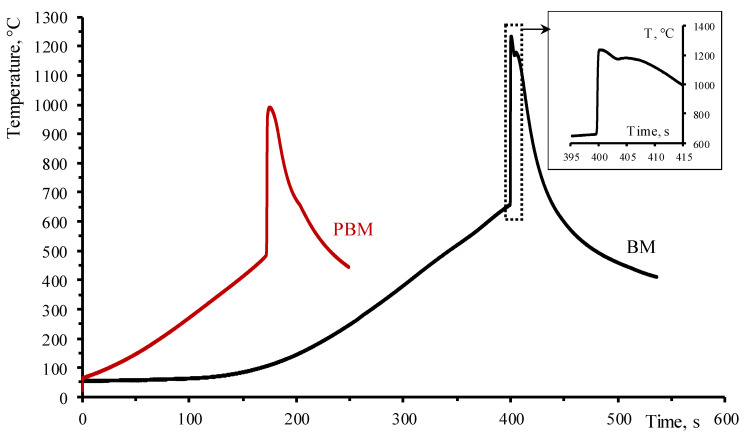
Thermograms of thermal explosion of MoAlB produced from the mixtures prepared in the BM and PBM.

Figure 4 shows the thermograms of thermal explosion of Mo-Al-B mixtures blended in the BM and PBM. The temperature profiles have a typical shape: a gradual rise in temperature at a rate of ~150 °C/min to the self-ignition point (T_ig_), followed by an abrupt increase in temperature to the final T_c_ due to exothermic reactions and a gradual decline in temperature in the post-combustion zone. For the mixture prepared in the BM, the T_ig_ is close to the melting point of aluminum (660 °C), while the maximum combustion temperature is 1234 °C. The presence of a small kink in the cooling section of the curve is probably associated with the secondary formation of MoAlB as a result of the interaction between the intermediate phases of MoB and Mo_3_Al_8_ with each other.

For the mixture prepared in the PBM, the temperature of the reaction initiation estimated according to the kink in the thermogram was T_ig_ = 490 °C, which was lower than that for synthesizing the mixture in the BM (heating rates being identical) (Figure 4). A fundamental distinction in the nature of interaction subsequent to MA in the planetary centrifugal mill is attributed to a multitude of interrelated factors, including the structural imperfections of the initial reagents and the reduced heterogeneity of the mixture. A reaction takes place between the components existing in the non-equilibrium state, increasing the structural imperfection of the material. Additional energy is supplied to it, thus enhancing diffusion activity. According to Kovalev et al. [50], it is the existence of non-equilibrium defects that ensures the implementation of the solid-phase mechanism of combustion of the mechanically activated mixtures. As a result, the temperature at which the reaction began was lower than that experienced during the formation of the non-activated mixture, which was heated at the same rate. The maximum combustion temperature was also lower than that of the mixture prepared in the BM and was 992 °C. Meanwhile, the thermogram contained no additional heat release peaks. A similar result, when the temperature of reaction initiation decreased after MA in the thermal explosion mode, was reported for the Ni-Al, Fe-Al, and Mg-B systems [50,51,52].

It is inferred from the measured combustion temperatures for mixtures prepared in the BM and PMM that aluminum is the only component that can melt in the combustion wave of this mixture. Therefore, the motive force of combustion in the Mo-Al-B system is the melting of aluminum, its capillary spreading over the surface of molybdenum and boron particles, and subsequent interaction through the melt.

The combustion of the MA mixture started at T_ig_ = 490 °C, being lower than the melting point of aluminum by 130 °C, which may also indicate that gas-phase reactions were intensified in the mixture after HEBM due to an increase in the oxygen content. MoB boride can be formed via the mechanism of gas-phase mass transfer of volatile MoO_3_ and B_2_O_2_ oxides toward B and Mo particles, respectively [53,54,55,56].

**Figure 5:**
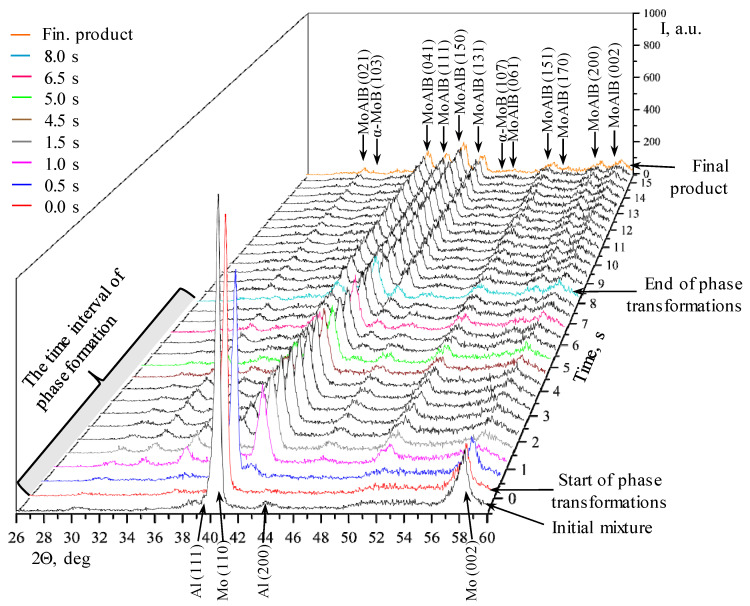
The series of XRD patterns of phase formation during thermal explosion of the Mo-Al-B mixture prepared in the BM.

**Figure 6:**
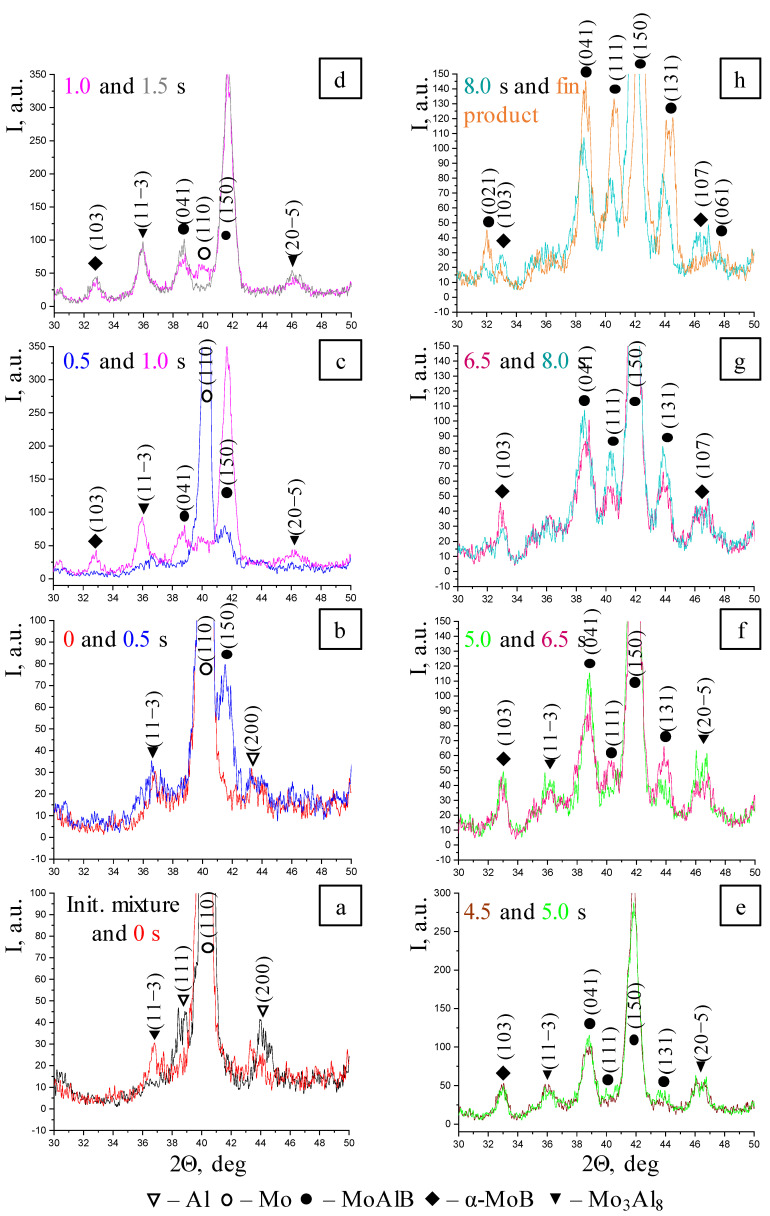
Selected series of XRD patterns recorded when heating the mixture prepared in the BM. (**a**)—Initial mixture and 0 s; (**b**)—0 s and 0.5 s; (**c**)—0.5 s and 1.0 s; (**d**)—1.0 s and 1.5 s; (**e**)—4.5 s and 5.0 s; (**f**)—5.0 s and 6.5 s; (**g**)—6.5 s and 8.0 s; (**h**)—8.0 s and final product.

Figure 5 shows a 3D projection of a consecutive series of XRD patterns recorded during the thermal explosion of the Mo-Al-B mixture prepared in the BM. The intensity of the diffraction lines changes during the thermal explosion of MoAlB, revealing the time parameters of phase emergence/disappearance. The time span for Figure 5 is 15 s; phase transformations are assumed to start at time point τ = 0 s (in Figure 3a, it corresponds to time point 24.0 s). For a comparative analysis, Figure 5 shows the XRD pattern of the initial Mo-Al-B mixture after processing it in the BM. The intensities of diffraction lines stop changing at τ > 8 s; i.e., all the phase transformations proceed for 8 s after the first phase changes started. In order to perform a more thorough analysis of phase formation stages, Figure 6 shows individual XRD patterns in the time interval of 0–8 s, at 2θ = 30°–50°. The diffraction pattern colors and times for Figure 5 and Figure 6 correspond to each other.

The first changes at the instant of thermal explosion compared to the initial mixture (Figure 6a) include a decline in intensity of the (110) diffraction line of Mo from 1400 to 1200 units and the (111) and (200) lines of Al, as well as the emergence of the (11-3) peak belonging to the Mo_3_Al_8_ intermetallic compound in the XRD pattern. After 0.5 s (Figure 6b), the intensity of the (110) line belonging to Mo continues to decrease (from 1200 to 900 units), and the first reflection of the MAB phase MoAlB (150) appears. The intensity of the (11-3) peak belonging to Mo_3_Al_8_ remains virtually unchanged; the XRD pattern still contains the (200) reflection of the initial Al. For the next 1.0 s (Figure 6c,d), the intensity of the (110) line corresponding to Mo decreases abruptly to the background level (from 900 to 30 units); the intensity of the (11-3) peak belonging to the Mo_3_Al_8_ intermetallic compound increases and (20-5) reflection appears, the intensity of the (150) peak corresponding to the MAB phase MoAlB increases, the (041) reflection appears, and molybdenum monoboride α-MoB is formed (the (103) peak). For the next 3.0 s, the phase composition remains unchanged.

The (111) and (131) reflections belonging to the MAB phase MoAlB appear in the XRD pattern 5.0 s after phase transformations began (Figure 6e). Next, after 1.5 s (Figure 6f), the intensities of the diffraction lines belonging to MoAlB increase, while the intensities of lines belonging to the Mo_3_Al_8_ intermetallic compound decrease. The reduced concentration of the Mo_3_Al_8_ intermediate phase indicates that it is involved in the final product formation reaction via the possible solid-phase reactions [17,26]:

Mo_3_Al_8(s)_ + 5MoB_(s)_ + 3B_(s)_ = 8MoAlB_(s)_ (1)

Mo_3_Al_8(s)_ + 5Mo_(s)_ + 8B_(s)_ = 8MoAlB_(s)_ (2)

Mo_3_Al_8(s)_ + 3B_(s)_ = 3MoAlB_(s)_ + 5Al_(l)_ (3)

If reaction (3) takes place, Al will interact with the intermediate MoB, yielding the MAB phase via reaction (4):

MoB_(s)_ + Al_(l)_ = MoAlB_(s)_ (4)

After another 1.5 s (Figure 6g), the peaks belonging to the Mo3Al8 intermetallic compound disappear completely, and the (107) reflection corresponding to the α-MoB boride phase emerges in the XRD pattern. The phase formation process is completed by 8.0 s (Figure 6h). The (021) and (061) diffraction lines belonging to MoAlB also appear as the sample cools, and all the peaks shift to smaller interplanar distances (larger angles).

To summarize, the subsequent stages of the MoAlB phase formation can be distinguished during the thermal explosion of the mixture prepared in the BM:

- Melting of Al and formation of the Mo_3_Al_8_ intermetallic compound, which subsequently reacts;
- Emergence of the MoAlB phase, as indicated by the highest-intensity (150) reflection;
- Formation of the α-MoB impurity phase after Mo_3_Al_8_ and MoAlB and its presence until the end of phase formation;
- After interaction starts, the peaks belonging to Al are present in the time interval of 0.5–1.0 s, while the diffraction lines corresponding to molybdenum remain for 1.5 s;
- All phase changes occur within 8 s, while the intermediate stage (“window”) lasts approximately 3 s.

**Figure 7:**
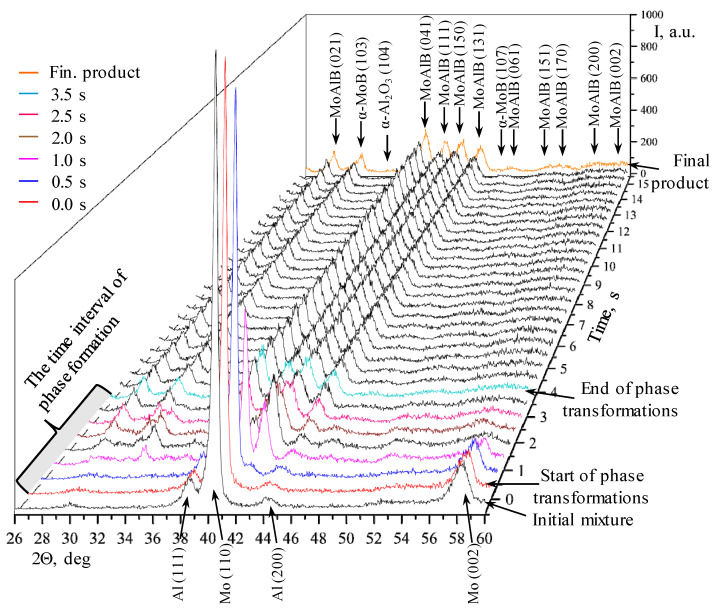
A series of XRD patterns of phase formation during thermal explosion of the Mo-Al-B mixture prepared in the PBM.

**Figure 8:**
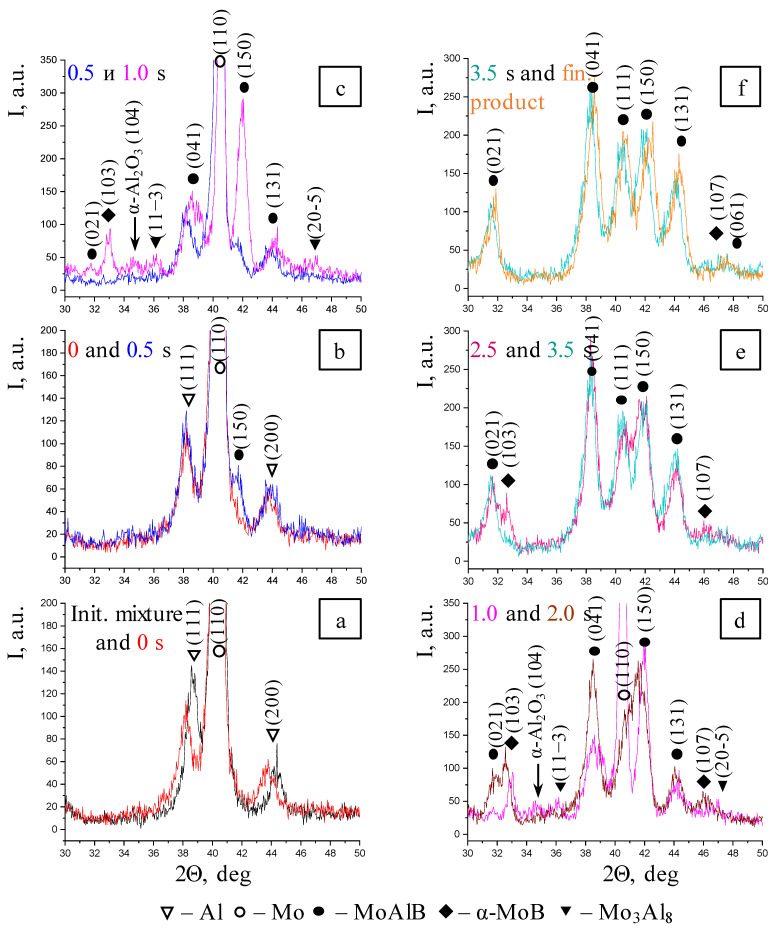
A selected series of XRD patterns recorded during the heating of the mixture prepared in the PBM. (**a**)—Initial mixture and 0 s; (**b**)—0 s and 0.5 s; (**c**)—0.5 s and 1.0 s; (**d**)—1.0 s and 2.0 s; (**e**)—2.5 s and 3.5 s; (**f**)—3.5 s and final product.

Figure 7 shows a 3D projection of a consecutive series of XRD patterns recorded during the thermal explosion of the Mo-Al-B mixture prepared in the PBM. Similar to Figure 5, phase transformations begin at time point τ = 0 s (in Figure 3b, it corresponds to time point 19.0 s). For conducting a comparative analysis, Figure 7 shows the XRD pattern of the initial Mo-Al-B mixture after processing in the PBM. In this case, the intensities of diffraction lines stop changing at τ > 3.5 s. Figure 8 shows individual XRD patterns in the time interval of 0–3.5 s at 2θ = 30°–50°. The diffraction pattern colors and times for Figure 7 and Figure 8 correspond to each other.

In the case of a thermal explosion of the sample fabricated from the mixture prepared in the PBM, phase formation begins earlier and occurs at a noticeably faster rate. Similar to the mixture prepared in the BM, the first changes are related to a decline in the intensities of diffraction lines of the initial components: Mo (110) from 2000 to 1900 units as well as Al (111) and (200) (Figure 8a). Meanwhile, no formation of intermediate compounds is detected. The first (150) reflection of the MAB phase MoAlB appears after 0.5 s; the (110) reflection belonging to Mo decreases to 1730 units, while the intensities of reflections for Al changes insignificantly (Figure 8b), thus indirectly indicating that MoAlB is formed in the system after MA via the solid-phase mechanism, without the formation of intermediate compounds.

By the end of the first second (Figure 8c), one can identify the (021), (041), (150), and (131) diffraction lines belonging to the MAB phase MoAlB, (11-3) and (20-5) diffraction lines corresponding to the Mo_3_Al_8_ intermetallic compound, the (103) peak belonging to molybdenum monoboride MoB, as well as the (104) reflection at 2θ = 34.6°, which presumably belongs to the oxide aluminum phase α-Al_2_O_3_, in the XRD pattern. By this time, the system still contains the unreacted molybdenum, for which the intensity of diffraction line is 710 units.

For the next 1.0 s (Figure 8d), the intensity of the (110) Mo peak decreases abruptly to 200 units, and the intensities of almost all the diffraction lines belonging to MoAlB and MoB boride increase, while the (11-3) and (20-5) reflections corresponding to the Mo_3_Al_8_ intermetallic compound disappear, thus indicating that it is involved in the formation of MoAlB via reactions (1)–(3).

The phase formation is completed 3.5 s after the commencement of phase transformations (Figure 8e). The X-ray lines belonging to MoB boride and the (104) reflection of aluminum oxide are rendered almost indistinct from the background. Only the (021), (041), (111), (150), and (131) peaks belonging to the MoAlB MAB phase remain in the XRD pattern. As observed in the case of the mixture prepared in the BM, all the peaks of the final products shift to smaller interplanar distances as the sample cools down (Figure 8f).

To summarize, the subsequent stages of the MoAlB phase formation can be distinguished during the thermal explosion of the mixture prepared in the PBM:

- Gas-phase mass transfer of volatile MoO_3_ and B_2_O_2_ oxides to the surface of B and Mo particles with the formation of the intermediate MoB phase, and in parallel with this, a solid-phase reaction between the initial components with the formation of the primary MoAlB phase corresponding (as in the case of the BM mixture) to the highest-intensity (150) reflection;
- Formation of all the major diffraction lines of the MAB phase within 2.5 s;
- Simultaneous formation of the MoB and Mo_3_Al_8_ impurity phases; the intermetallic compound disappears at the post-reaction stage;
- After the interaction begins, the peaks belonging to Al are present in the time interval of 0.5–1.0 s; the lines corresponding to molybdenum are observed in the XRD pattern for 2.0 s;
- All the phase transformations occur within 3.5 s.

**Figure 9:**
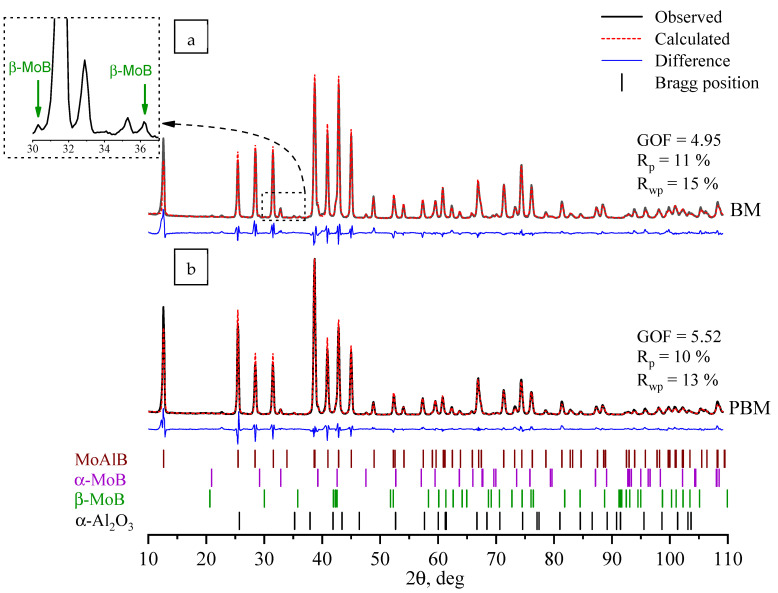
XRD patterns of the synthesis products obtained from the mixtures subjected to treatment in the BM (**a**) and PBM (**b**).

The results of the XRD phase analysis of the synthesis products obtained from the mixtures treated in the BM and PBM are presented in Table 1 and Figure 9. The calculated values of the divergence factors of the experimental and calculated diffraction patterns were R_p_ = 10–11%, R_wp_ = 13–15%, and GOF = 4.95–5.52. The method employed to prepare the reaction mixture was found to have a negligible impact on the phase composition of the final SHS products. MoAlB crystallizing in the orthorhombic system was the major phase (92–94%). The unit cell parameters of the MAB phase were virtually identical. Furthermore, the synthesis products contained approximately 3–5% of the low-temperature tetragonal modification of α-MoB that was detected through time-resolved X-ray diffraction, as well as 2–3% of the α-Al_2_O_3_ aluminum oxide phase. The sample produced from the green mixture treated in the BM additionally contained 1% of the high-temperature orthorhombic β-MoB modification.

**Figure 10:**
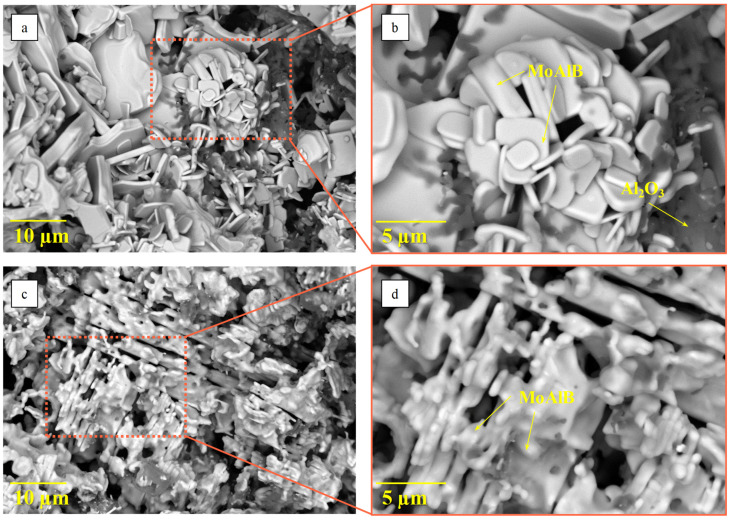
The morphology of the synthesis products obtained from the mixtures subjected to treatment in the BM (**a**,**b**) and PBM (**c**,**d**).

Figure 10 shows the morphology of the synthesis products obtained from the reaction mixtures prepared in the BM and PBM. Lamellar grains of the MAB phase MoAlB, 0.4 µm thick and ~2–10 µm long, are the main structural components of the ceramics. The sample produced from the PBM mixture has a finer-grained structure. Dark agglomerated grains sized up to 1 µm residing in the intergrain pores belong to the Al_2_O_3_ phase.

Our findings, with the stages of MoAlB formation identified, show good agreement with the data reported in ref. [37], which reports an in situ study on the mechanism of MoAlB synthesis from element powders using neutron diffraction. The key differences are that stages corresponding to the formation of the intermediate MoAl_12_ phase and the high-temperature orthorhombic modification of β-MoB instead of α-MoB are reported in ref. [37]. These differences are probably related both to the fact that neutrons are characterized by a deeper penetration ability and the crystal structure can be studied over a broad 2θ range = 15°–120° and to the different heating rates used in the experiments—~5 °C/min and ~500 °C/min—when heating the sample in a tube furnace and an induction furnace, respectively. In our experiments, temperature was increased to the autoignition point at a rate of ~150 °C/min.

Hence, our investigation demonstrates that single-phase MoAlB ceramics can be synthesized from mixtures prepared in the BM and PBM in the thermal explosion mode. Further research will focus on the synthesis of compacted single-phase ceramics by combining SHS (for fabricating the precursor powder) and hot-pressing (HP) as well as on the effect of the type of powder mixing on the structural phase state, mechanical properties, and high-temperature oxidation resistance of consolidated materials.

## 4. Conclusions

The effect of the method used to prepare the Mo-Al-B reaction mixture on the mechanism of formation of the MAB phase MoAlB during thermal explosion was investigated using time-resolved X-ray diffraction. For the mixtures prepared in the BM and PBM, the measured maximum combustion temperatures indicated that aluminum melted during synthesis, and that molybdenum and boron interacted through the melt. Both cases showed that Mo_3_Al_8_ and α-MoB intermediate compounds were formed in the combustion front, and the intermetallic phase was consumed to form the MAB phase. The formation of MoAlB was observed to commence with the highest-intensity (150) reflection; for the mixture prepared in the BM, the MAB phase was formed subsequent to the melting of Al and the formation of the Mo_3_Al_8_ intermetallic phase. For the mixture prepared in the PBM, the initial interaction stage started with the direct solid-phase reaction of MoAlB formation. The study demonstrated that, after interaction initiation, the initial Al remained in the reaction mixture for 0.5–1.0 s, while molybdenum was completely reacted for 1.5–2.0 s. All the phase transformations occurred within ~8 s and ~3.5 s for the mixtures prepared in the BM and PBM, respectively.

## Figures and Tables

**Table 1:** Phase composition of the synthesized samples.

Phase	MoAlB			α-MoB	β-MoB	α-Al_2_O_3_
Space group	Cmcm			I4_1_/amd	Cmcm	R-3c
Crystal system	Orthorhombic			Tetragonal	Orthorhombic	Hexagonal
Phase parameters	Wt., %	Unit cell parameters, Å	Unit cell volume, Å^3^	Wt., %	Wt., %	Wt., %
SHS products from BM mixture	92	a = 3.211	139.22	5	1	2
		b = 13.977				
		c = 3.102				
SHS products from PBM mixture	94	a = 3.213	139.41	3	-	3
		b = 13.979				
		c = 3.104				

## Data Availability

Data is contained within the article.
